# Interrelationships among an Inflammatory Diet and *Helicobacter pylori* infection on Cognition in older adults

**DOI:** 10.1002/alz.088607

**Published:** 2025-01-09

**Authors:** Yi‐Chun Chou, Ming‐Lun Han, Tina H.T. Chiu, Meei‐Shyuan Lee, Jeng‐Min Chiou, Yen‐Ching Chen, Jen‐Hau Chen

**Affiliations:** ^1^ National Taiwan University Hospital, Taipei City, Taipei Taiwan; ^2^ Fu Jen Catholic University, New Taipei City, Taipei Taiwan; ^3^ School of Public Health, National Defense Medical Center, Taipei City, Taipei Taiwan; ^4^ Institute of Statistics and Data Science, National Taiwan University, Taipei City, Taipei Taiwan; ^5^ College of Public Health, National Taiwan University, Taipei City, Taipei Taiwan; ^6^ College of Medicine, National Taiwan University, Taipei City, Taipei Taiwan

## Abstract

**Background:**

The role of diet in modulating inflammation and its potential impact on cognition has raised attention. Additionally, recent research has addressed the disruption of the gut‐brain axis in dementia development. This study aims to explore how the interactions between inflammatory diets and *Helicobacter pylori* (*Hp*) infection affect cognitive domains in older adults.

**Method:**

This eight‐year prospective cohort study recruited 511 non‐demented community‐dwelling older adults (age≥65 years) in the Taipei Metropolitan area from 2011 to 2019. At baseline, we assessed dietary inflammatory potential using the Empirical Dietary Inflammatory Index (EDII) through a 44‐item semi‐quantitative food frequency questionnaire. The level of *Hp* immunoglobulin G (IgG) was determined. Global and domain‐specific cognition were evaluated at baseline and biennial follow‐ups. A generalized linear mixed model was used to examine the association between EDII and cognition adjusting for important covariates. Stratified analyses by *Hp* seropositivity were conducted, and its interactions with EDII were tested.

**Result:**

The average age of the study participants was 72.6 years with women accounting for 53%. Each additional increment in EDII (i.e., a more pro‐inflammatory diet) was associated with poor memory performance (logical memory‐immediate free recall: *β* = ‐0.69 and delayed free recall: *β* = ‐0.72). After stratification, one unit increment in EDII was associated with poor performance of global cognition (MoCA‐T: *β* = ‐1.68) and memory (logical memory‐immediate free recall: *β* = ‐1.07; delay free recall: *β* = ‐1.02) among participants with *Hp* seropositivity (*Hp* IgG ≥1.1 U/mL). Additionally, significant interactions were found between EDII and *Hp* seropositivity in the domains of executive function (*p_interaction_* = 0.08) and attention (*p_interaction_* = 0.02).

**Conclusion:**

Our findings suggest that an inflammatory diet interacts synergistically with *Hp* infection, contributing to poor cognition. Preventing cognitive decline necessitates a holistic approach including healthy eating and *Hp* eradication.

